# Pathway Activation Analysis for Pan-Cancer Personalized Characterization Based on Riemannian Manifold

**DOI:** 10.3390/ijms25084411

**Published:** 2024-04-17

**Authors:** Xingyi Li, Jun Hao, Junming Li, Zhelin Zhao, Xuequn Shang, Min Li

**Affiliations:** 1 School of Computer Science, Northwestern Polytechnical University, Xi’an 710072, China; xingyili@nwpu.edu.cn (X.L.); haojun@mail.nwpu.edu.cn (J.H.); shang@nwpu.edu.cn (X.S.); 2 School of Software, Northwestern Polytechnical University, Xi’an 710072, China; ljmsup@mail.nwpu.edu.cn (J.L.); zz263943@mail.nwpu.edu.cn (Z.Z.); 3 School of Computer Science and Engineering, Central South University, Changsha 410083, China

**Keywords:** pathway activation, Riemannian manifold, pan-cancer analysis, personalized characterization, pathway biomarkers

## Abstract

The pathogenesis of carcinoma is believed to come from the combined effect of polygenic variation, and the initiation and progression of malignant tumors are closely related to the dysregulation of biological pathways. Quantifying the alteration in pathway activation and identifying coordinated patterns of pathway dysfunction are the imperative part of understanding the malignancy process and distinguishing different tumor stages or clinical outcomes of individual patients. In this study, we have conducted in silico pathway activation analysis using Riemannian manifold (RiePath) toward pan-cancer personalized characterization, which is the first attempt to apply the Riemannian manifold theory to measure the extent of pathway dysregulation in individual patient on the tangent space of the Riemannian manifold. RiePath effectively integrates pathway and gene expression information, not only generating a relatively low-dimensional and biologically relevant representation, but also identifying a robust panel of biologically meaningful pathway signatures as biomarkers. The pan-cancer analysis across 16 cancer types reveals the capability of RiePath to evaluate pathway activation accurately and identify clinical outcome-related pathways. We believe that RiePath has the potential to provide new prospects in understanding the molecular mechanisms of complex diseases and may find broader applications in predicting biomarkers for other intricate diseases.

## 1. Introduction

Carcinoma is driven by multiple factors and the underlying molecular mechanisms of cancer pathogenesis are complex; it is one of the most lethal diseases in the world. Genome-wide association studies (GWASs) and next-generation sequencing technologies [1] have continuously provided insights into the genetics of cancers, and varied single-gene biomarkers have been identified to play an important role in the early diagnosis, prognosis, and efficacy evaluation of cancers [2,3]. Canonically, most widely used methods are dedicated to finding differentially expressed genes [4,5]. However, the selection process is subjective, variations among samples are astronomical, and the functional understanding of the pathogenesis of carcinoma is intractable [6]. Meanwhile, with the heterogeneity of cells in tissues and the genetic heterogeneity between patients with complex diseases, most anticancer drugs are only effective in subgroups of patients [7]; it is urgent to develop personalized cancer treatments [8,9,10]. Pathway-based individualized analysis can overcome these challenges by using robust, aggregate features to reveal the molecular mechanisms of complex diseases [11].

Pathways are a series of biological activities among molecules in cells and are expressive of the biological processes within cells, such as metabolism, signaling, and growth cycles, which will lead to some alteration or obtain some products [12]. Misregulation of activation for several important pathways has been found to be associated with cancer initiation and progression [13,14,15,16], identifying the pathways involved in the occurrence and progress of cancer and quantifying their dysregulation are the imperative part of understanding the process of malignancy. With the development of high-throughput technologies, a large amount of biological data has been generated, which has produced a rich set of pathway databases [17,18,19,20,21]. Therefore, it is of great significance to use bioinformatics tools to mine pathways related to the pathogenesis of cancers based on high-throughput data for the diagnosis and treatment.

Current quantitative pathway-centric measures have been proposed to evaluate the pathway activation and identify the dysregulated pathways in cancer initiation and progression. Some works extract critical features from expression values of genes belonging to pathways [10,22,23,24]. Some works consider the intrinsic structures of pathways; they calculate personalized pathway activation scores based on the topological information of pathways [11,25]. Many of these methods evaluate the pathway deregulation scores based on the Euclidean space, and although they are constantly improving the classification ability of diseases, they are still less than satisfactory in some analyses. They use Euclidean space since it is easy to be implemented and applied in practice. However, if the data samples do not lie on Euclidean space, the rationality and effectiveness of these methods cannot be guaranteed, since the differences calculated on the Euclidean space cannot represent the real geometrical relations among samples.

In order to further improve the classification accuracy, we have firstly developed a Riemannian manifold-based method, RiePath, to evaluate the pathway deregulation scores for each patient on the tangent space of the Riemannian manifold. It can not only convert gene-level expression information to pathway-level deregulation information, so as to achieve the dimensionality reduction, but also has the potential to identify essential biological pathways as biomarkers.

We compare RiePath to other feature engineering algorithms; the results show that our method can not only obtain higher clustering accuracy in the discrimination of normal and tumor samples and reproducibility, but also effectively capture the potential prognostic-related pathway biomarkers, which have the functional interpretability to explore the biological mechanism of carcinoma from a molecular level.

## 2. Results

### 2.1. Performance Comparison with Other Feature Engineering Methods

For further comparison, we first build a scheme from The Cancer Genome Atlas (TCGA, https://portal.gdc.cancer.gov/, accessed on 7 April 2024) Breast invasive carcinoma (BRCA) cohort to demonstrate the performance of our approach, as well as compare it with other state-of-the-art feature engineering methods. We first randomly select 50 samples from disease and normal samples. Then, the pathway activation matrix is calculated by each feature engineering algorithm according to the RNA-seq data of the randomly selected samples, where each row represents a pathway and each column represents a sample. Next, limma R package is used to perform differential expression analysis and select the top 10 differential expression pathways based on the adjusted *p* values. Subsequently, we utilize the hierarchical clustering algorithm to divide the 100 samples into two classes and compare the clustering results and sample labels through the adjusted rand index (ARI) evaluation index. The above steps are repeated 50 times in each method.

To demonstrate the discrimination effectiveness of pathway activation calculated by RiePath, We choose four representative pathway activation measurement algorithms: CORGs [10], GSVA [26], PLAGE [27], and ssGSEA [28], and the compared methods are implemented with default parameters.

CORGs defines a subset of genes in a pathway named as condition-responsive genes, which are considered to play a crucial role in each pathway. For each pathway, the pathway activity score is evaluated by a combined z-score derived from the expression of condition-responsive genes.

GSVA is a gene set variation analysis method that calculates the variation in gene set enrichment over a sample population as the pathway activation. GSVA sorts genes based on the Kernel estimation of the cumulative density function of genes in the gene set, and calculates a Kolmogorov–Smirnov-like rank statistic for each gene set.

PLAGE can analyze the dysregulation level of a pathway by estimating the pathway activity based on the first eigenvector in the singular value decomposition of gene expression data.

ssGSEA is a single sample gene set enrichment analysis method, and the enrichment score is computed by the integration of the difference between weighted Empirical Cumulative Distribution Functions (ECDFs) of the genes in the signature and the ECDFs of the remaining genes.

**Figure 1:**
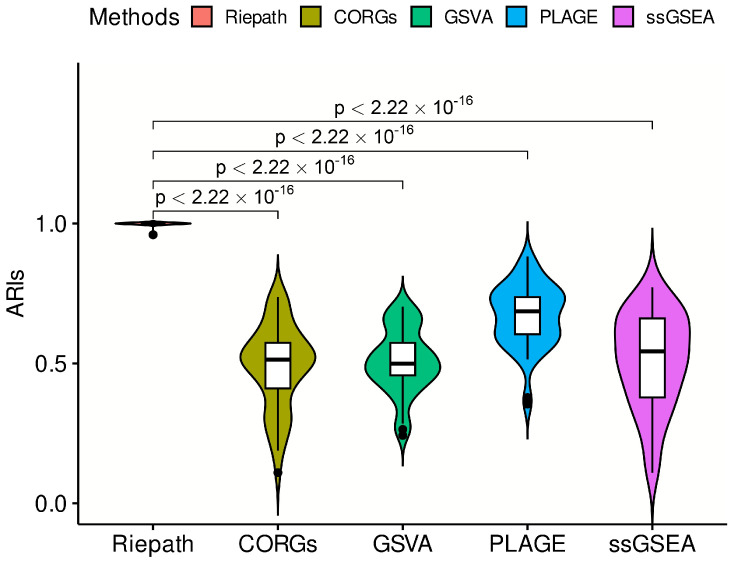
Hierarchical clustering comparison measured by ARI index.

The hierarchical clustering results measured by ARI index for clustering accuracy are shown in Figure 1. Higher ARI values means that samples with the same label are better clustered into one class, which can also prove that the feature engineering method of calculating the pathway activation can better capture the deviation of patients from the signature of healthy samples. As expected, the pathway dysregulation scores calculated by RiePath have more stable and better clustering performance than the other compared methods, and the pairwise comparison between RiePath and the other four methods using the *t* test can prove that the differences are statistically significant.

Then, we compare the reproducibility of RNA-seq data to evaluate the how well gene-level sample differences are kept at the pathway level [12,29]. The reproducibility score (RS) is defined as the reciprocal of the weighted average of mean squared error (MSE):

(1) RS=N2∑1≤a,b≤N{Sim(Xa,Xb)−Sim(Aa,Ab)}2

where $X=[X_{1},\ldots ,X_{N}]$ is the gene expression matrix, $A=[A_{1},\ldots ,A_{N}]$ is the pathway activation matrix, and Sim denotes the cosine similarity.

The smaller the distance between gene expression and pathway activation values, the greater the RS value is, which demonstrates that the sample space of the inferred pathway activation scores approaches that of the original gene expression. This is due to the fact that pathways only contain a small subset of genes, accounting for approximately one-third of all genes in the gene expression. After calculating the pathway activation scores, repeatability characteristics can measure how well the distance between samples in terms of pathway activation can maintain the characteristics of the original data (i.e. all genes). As shown in Table 1, RiePath obtains the highest RS value compared to other feature engineering methods on each cancer type dataset, which means that our method is effective in retaining the characteristics of the original transcriptome data.

### 2.2. The Identification of Dysregulated Pathways

For each pathway, all the tumor samples are firstly divided into two categories, dysregulated or near-normal samples, based on the mean and standard deviation of the normal samples’ RiePath scores. If one tumor sample is classified as ‘dysregulated’ on this pathway, it means that the gene expression level of this tumor sample on this pathway severely deviates from that of the normal level, and this pathway is classified as ‘dysregulated’ on this tumor sample correspondingly. Otherwise, the tumor sample is classified as ’near-normal’ on this pathway and this pathway is classified as ’near-normal’ on this tumor sample accordingly.

**Figure 2:**
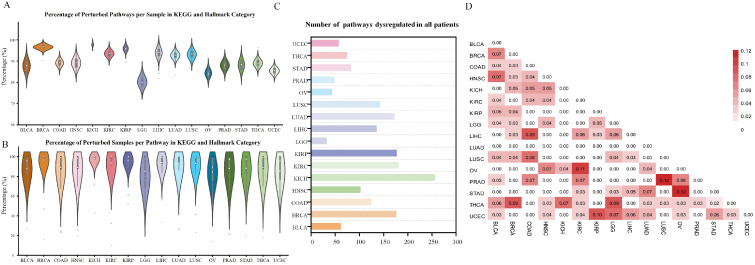
Survey of the important pathways in the 16 cancer types. (**A**) The violin plot of percentage of dysregulated pathways on each sample in KEGG and Hallmark category. (**B**) The violin plot of percentage of dysregulated samples on each pathway in KEGG and Hallmark category. (**C**) The number of pathways dysregulated in all patients. (**D**) The similarity of the overlapping prognostic pathway biomarkers identified in 16 cancer types using the Jaccard index.

For complex diseases like cancers, several vital pathways are often dysregulated simultaneously; the key to consider is how to effectively identify the dysregulated pathways for a specific tumor sample and whether there are differences among different cancer categories. Therefore, we comprehensively investigate the Kyoto Encyclopedia of Genes and Genomes (KEGG, https://www.genome.jp/kegg/, accessed on 7 April 2024) [17] pathways and Molecular Signatures Database (MSigDB, http://www.gsea-msigdb.org/gsea/msigdb, accessed on 7 April 2024) [30] Hallmark gene sets. Figure 2A shows the violin plot of percentage of dysregulated pathways on each sample in KEGG and Hallmark category; for the 16 tumor types, it can be seen that there are obvious differences. Kidney chromophobe (KICH) shows the highest proportion of dysregulated pathways, which is 98%. Conversely, Low-grade gliomas (LGG) shows the lowest proportion (79%). Interestingly, the percentages of dysregulated samples on each pathway in the KEGG and Hallmark category have less variation among the 16 kinds of cancer, as shown in Figure 2B. Moreover, for each of the 16 tumor categories, we also make a survey of the number of pathways dysregulated in all patients. As shown in Figure 2C, there are 344 pathways in the KEGG and Hallmark category, and the number of pathways perturbed in all disease samples are highest in KICH (256 pathways), and conversely, the least in LGG (32 pathways).

### 2.3. The Identification of Prognostic Pathway Biomarkers

As the indicators to determine the progression and recurrence of tumors, prognostic biomarkers play an important role in cancer research. In this study, we propose RiePath to calculate the pathway activation of each pathway for each patient, and RiePath is applied in 344 pathways to 7291 disease samples across 16 TCGA cancer types. For each kind of cancer, we identify candidate prognostic pathway biomarkers using Kaplan–Meier analysis and setting the significance threshold of log-rank *p* value less than 0.05, which is consistent with the threshold used in many studies to identify prognostic biomarkers [7,31].

Supplementary Table S1 lists the prognostic pathway biomarkers identified based on the RiePath scores across the 16 cancer types. A total of 164 pathways are identified as promising prognostic pathway biomarkers across 16 cancer types, and the number of prognostic pathway biomarkers among different kinds of cancer are imbalanced. Most of the important pathways identified based on our method are mainly concentrated in Liver infiltrate hepatocellular carcinoma (LIHC) and Thyroid carcinoma (THCA), while a small number occur in the two kidney cancer types: KICH and Kidney renal papillary cell carcinoma (KIRP). Moreover, we introduce the Jaccard index to measure the similarity of the overlapping biomarkers identified in 16 cancer types from a pan-cancer perspective, and the Jaccard similarity index of two sets of prognostic pathways for each pair of cancer types is defined as follows:

(2) $$J\left(u,v\right)=\frac{\left|u\cap v\right|}{\left|u\cup v\right|}=\frac{\left|u\cap v\right|}{\left|u\right|+|v|-|u\cap v|}$$

where *u* and *v* are the prognostic pathway biomarker sets of every two cancer types. If *u* and *v* are the same set, that is, the set of prognostic pathway biomarkers for the same cancer type, $J\left(u,v\right)=0$. We observe that the overlapping prognostic pathway biomarkers shared by two cancer types in the KEGG and Hallmark category are very few (Figure 2D), meaning that the majority of prognostic pathway biomarkers identified in each cancer type are specific rather than shared, reflecting the diversity of human malignancies. This result is consistent with the conclusion presented in [7].

### 2.4. The Selection of Prognostic Pathway Biomarkers

Among the prognostic pathway biomarkers identified based on RiePath, there are many pathways closely related to the occurrence and progression of cancer, especially signaling pathways. Mutations in cancer cell genomes affect signaling pathways that play key roles in cell growth, proliferation, angiogenesis, survival, apoptosis, and metastasis. Activation of these pathways will result in the upregulation of transcription factors that induce epithelial–mesenchymal transition in cells [32]. Several signaling pathways are critical for the embryonic development, which plays a key role in tumor progression and changes in response to the therapy in different cancers [33].

In BRCA, several signaling pathways are identified as prognostic pathway biomarkers based on RiePath scores from the KEGG database. The identification of the cAMP signaling pathway, PI3K/AKT signaling pathway, and VEGF signaling pathway have been verified by many studies to play an important role in the occurrence and development of breast cancer and are closely related to endocrine therapy resistance in the later period of breast cancer [34,35,36,37,38]. For example, it has been recognized that the elevated levels of intracellular cAMP will stimulate the growth of the normal human breast epithelial cells in culture [39]. Several studies have demonstrated that cAMP inhibits the growth of established breast cancer cell lines and breast cancer cells in primary culture [39,40,41]. In addition to the KEGG pathway database, we also identify HALLMARK_MTORC1_SIGNALING as the biomarker for BRCA. MTORC1 signaling has been supported in a previous study where *PIK3CA* mutations are associated with gene signature of low MTORC1 signaling in estrogen receptor-positive breast cancer, and *PIK3CA* mutations are one of the most common genetic aberrations in breast cancer [37,42].

**Figure 3:**
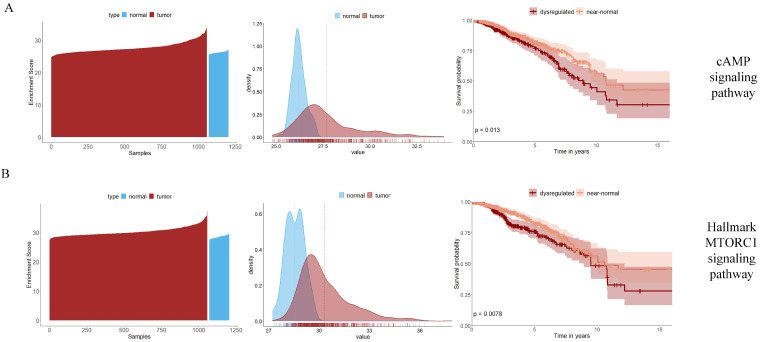
cAMP and Hallmark MTORC1 signaling pathways identified by RiePath scores in BRCA. (**A**) cAMP signaling pathway. (**B**) Hallmark MTORC1 signaling pathway. The waterfall and density plots of RiePath scores in tumor and normal samples are shown in the first and second columns, and the Kaplan–Meier plots indicate the significant survival difference for the dysregulated and near-normal patients in the two pathways.

Subsequently, we analyze two signaling pathways: “cAMP signaling pathway” and “HALLMARK_MTORC1_SIGNALING”. From the waterfall plots and density plots in Figure 3A,B (first column shows the waterfall plots and second column shows the density plots), there is a significant difference between the overall RiePath values of the disease samples and that of the normal samples. Meanwhile, the log-rank test in the survival analysis (third column of Figure 3A,B) shows significance (*p* value < 0.05) on these two pathways, which indicates that these are two prognostic pathway biomarkers. Other visual summaries of signaling pathways identified by RiePath scores in the remaining cancer types are shown in Supplementary Figures S1–S15.

## 3. Discussion

Gene-level information has been widely used in the studies of complex diseases, especially in cancer research. But they are often sensitive to noise and low repeatability. Integrating gene expression and pathway information can obtain an aggregate and biologically relevant representation, which quantifies the dysregulation of pathways and has the potential to identify essential pathways involved in the complex diseases.

In this study, we have proposed a Riemannian manifold-based method, RiePath, to evaluate the pathway dysregulation scores for each individual patient on the tangent space of the Riemannian manifold. RiePath converts high-dimensional gene-level expression information into relatively low-dimensional pathway-level dysregulation information, generating a compact and biologically relevant representation. This is the first attempt to apply Riemannian manifold theory to infer the degree of dysregulation at the pathway level so as to understand the molecular mechanisms of diseases. We apply the proposed method to the analysis of 16 cancer types. The results demonstrate that RiePath can not only have higher performance, but also effectively capture the potential prognostic pathway biomarkers with functional interpretability, which can not only provide better understandings of the mechanisms of cancer progression and drug therapy, but is also critical to the improvement of treatments.

RiePath is a clinically relevant predictive method that measures pathway dysregulation scores using pathway and gene expression data. It can also be extended to any other kind of data with known pathway assignments. Meanwhile, the development of single-cell sequencing technologies provides the possibility to reveal the mechanism of complex diseases at the cellular level [43,44]. RiePath is data-based and the pathway dysregulation is context-specific, so it also has the potential to be extended to single-cell study in future work.

## 4. Materials and Methods

### 4.1. Data Collection

**Figure 4:**
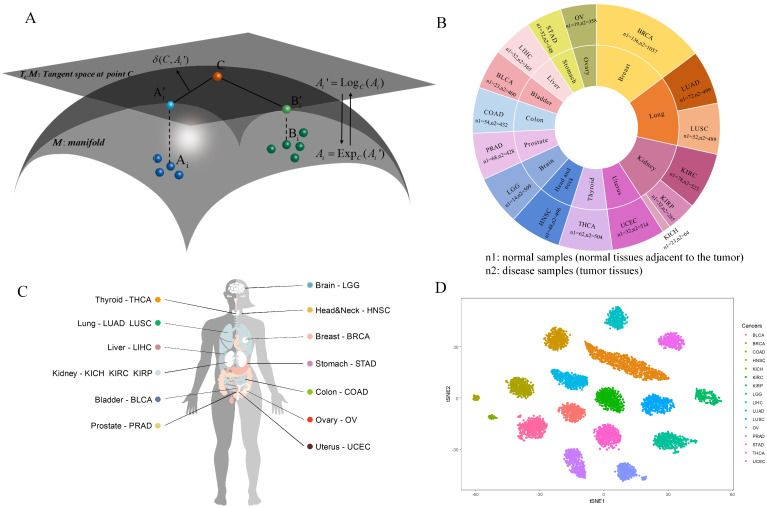
Overview of the RiePath algorithm. (**A**) Illustration of the Riemannian manifold and tangent space at point C. (**B**) The number of samples in each cancer type. (**C**) The 16 TCGA cancer types that are analyzed in the pan-cancer study. (**D**) t-SNE data visualization of the RiePath scores from all patients with tumor tissues of the 16 cancer types.

Gene expression profiles and corresponding clinical information used in this study are generated by TCGA datasets. The high-throughput sequencing data of TCGA are downloaded from the UCSC Xena browser (https://xenabrowser.net/, accessed on 7 April 2024), and we obtain the Fragments per Kilobase of transcript per Million mapped reads (FPKM) processed normalized gene expression profiles from each of cancer types. All the data are log2 transformed. We only keep the first vital in a sequence of samples. Considering that RiePath and some comparison algorithms need to use normal samples as the background group, we download the tumor-adjacent tissue samples as normal samples. Meanwhile, cancer types with more than 10 normal samples and disease samples will be used in the pan-cancer study to ensure the sufficient sample sizes. Totally, 16 TCGA projects (BLCA, BRCA, COAD, LGG, HNSC, PRAD, THCA, KIRC, KIRP, KICH, LIHC, LUAD, LUSC, OV, STAD, UCEC) meet the requirements, as shown in Table 2 and Figure 4B,C.

Pathways come from the KEGG database and MSigDB Hallmark gene sets. For the pathway data from KEGG database, the KGML (KEGG XML) files are parsed into graph models maintaining all essential pathway attributes using the KEGGgraph package [45]. For a pathway, it contains nodes and edges; a node represents a gene, and an edge is the interaction between two genes. Considering the subsequent analysis, genes not existing in the RNA-seq gene expression data, and the corresponding edges, are discarded. Therefore, 294 pathways with 4156 nodes and 17,349 edges are obtained. Meanwhile, 50 biological pathways from the MSigDB Hallmark signature collection are also considered into this study. In total, there are 344 pathways used in our analysis.

### 4.2. Space of Symmetric Positive Definite (SPD) Matrices

Given a gene expression matrix $X_{i}\in R^{N\times E}$ with *N* genes and *E* samples, the space of symmetric matrices can be denoted as follows:

(3) $$S\left(N\right)=\left\{S\in R^{N\times N},S^{T}=S\right\}$$

and the space of positive-definite matrices can be denoted as:

(4) $$P\left(N\right)=\left\{P\in R^{N\times N},u^{T}Pu>0,\forall u\in R^{N}\right\}$$

the space of SPD matrices is denoted as:

(5) $$SPD\left(N\right)=S\left(N\right)\cap P\left(N\right)$$

The space of SPD matrices lie on a differentiable Riemannian manifold *M* (Figure 4A) with the dimensionality of N(N+1)/2 [46].

For two spatial covariance matrices (two points) $A_{i}\in R^{N\times N}$ and $C\in R^{N\times N}$ on the manifold, the Riemannian distance $\delta_{R}\left(C,A_{i}\right)$ between them is defined as:

(6) $$\delta_{R}\left(C,A_{i}\right)={∥log(C^{-1}A_{i})∥}_{F}=\sqrt{\sum_{n=1}^{N}log^{2}\left(\lambda_{n}\right)}$$

where log(·) denotes the logarithmic operator, $\left|\right|\cdot {\left|\right|}_{F}$ is the Frobenius norm of a matrix, and ${\left\{\lambda_{n}\right\}}_{n=1}^{N}$ the real strictly positive eigenvalues of $C^{-1}A_{i}$. This distance represents the length of unique shortest curve (called geodesic) connecting the two points in the Riemannian manifold [47].

### 4.3. Riemannian Tangent Space

The derivatives of each point in the Riemannian manifold can form a tangent space $T_{C}M$, and it consists of a set of tangent vectors [48,49]. The tangent space, as a Euclidean space, is an important space in the analysis of a Riemannian manifold. The logarithmic mapping operator that can project a point on the manifold to the tangent space at point *C* is defined as:

(7) $${A_{i}}^{\prime }={Log}_{C}(A_{i})=C^{\frac{1}{2}}log(C^{-\frac{1}{2}}A_{i}C^{-\frac{1}{2}})C^{\frac{1}{2}}$$

The inverse operation is the exponential mapping that projects a tangent element back to the original manifold:

(8) $$A_{i}={Exp}_{C}\left({A_{i}}^{\prime }\right)=C^{\frac{1}{2}}exp\left(C^{-\frac{1}{2}}{A_{i}}^{\prime }C^{-\frac{1}{2}}\right)C^{\frac{1}{2}}$$

where log(·) and exp(·) are the logarithm and exponential of a matrix, respectively. They are operators that map one-to-one between Riemannian manifold and tangent space. Figure 4A illustrates this process.

At each point of the manifold, a scalar product can be defined in the associated tangent space. The tangent space, as a Euclidean space, is an important space in the analysis of a Riemannian manifold. The tangent space $T_{c}M=\left\{Log_{C}\left(A_{i}\right),A_{i}\in SPD\left(N\right)\right\}$ at point *C* is a space of symmetric matrices and there are only N(N+1)/2 independent elements. The minimal representation of the tangent space can be found as a vector space [50]:

(9) $$T_{c}M=\left\{{A_{i}}^{\prime }=upper\left(C^{-\frac{1}{2}}Log_{C}\left(A_{i}\right)C^{-\frac{1}{2}}\right)\in R^{N(N+1)/2}\right\}$$

where the operator upper(·) is to keep the upper triangular portion of symmetric matrix and vectorize it.

The distance between point *C* and point $A_{i}$ on the Riemannian manifold *M* can be measured as the Euclidean distance from point *C* and point ${A_{i}}^{\prime }$ the tangent space at point *C* [49]:

(10) $$\delta \left(C,{A_{i}}^{\prime }\right)=\left|\right|{A_{i}}^{\prime }-0{\left|\right|}_{2}$$

where ${A_{i}}^{\prime }\in T_{C}M$ the vector in tangent space corresponding to $A_{i}\in M$. The tangent space $T_{C}M$ of Riemannian manifold *M* at point *C* is shown in Figure 4A.

### 4.4. Pathway Dysregulation Scores

RiePath is a novel computational algorithm to evaluate pathway deregulation scores for each individual sample based on Riemannian manifold, enabling the accurate evaluation of pathway activation and the identification of the powerful pathway biomarkers for the pan-cancer personalized characterization across 16 cancer types.

RiePath aligns pathways and gene expression data to project samples into the space of SPD matrices, which evaluates the degree of pathway dysregulation from normal to disease. To estimate the pathway dysregulation score, we utilize the expression values of genes belonging to each pathway. There are three steps to measure the pathway deregulation scores: (1) The covariance matrix of the group of normal samples is calculated. The covariance matrix is an SPD matrix, which lies on a Riemannian manifold and will be projected as a point, that is, the reference point *C* in Figure 4A. (2) An individual patient will be added into the group of healthy samples, and we will obtain a point whose distance from the reference point represents the perturbation of the patient. Repeat this step until we obtain all the perturbed points in the Riemannian manifold based on all the patients. (3) We map the perturbed points in the Riemannian manifold to the tangent space at the reference point *C* and calculate the distance between reference point and each perturbed point. This distance is considered to be the extent to which each pathway deviates from the normal in each patient, that is, the extent of dysregulation. After calculating the distances between all perturbed points and reference point for all the pathways, the high-dimensional gene expression matrix will be converted into a low-dimensional matrix for pathways and patients. The t-SNE data visualization of RiePath scores for all tumor samples across the 16 cancer types is shown in Figure 4D. It indicates that tumor samples from the same cancer type are compactly clustered together and different types of tumor samples are separated from each other well, which means that the pathway activation values obtained by our algorithm can effectively distinguish samples of different cancer types. The pathway activation analysis can provide effective assistance in identifying cancer-specific pathway biomarkers for precision medicine.

## 5. Conclusions

In this study, we test a novel method named RiePath, which analyzes the pathway activation and identifies coordinated patterns of pathway dysregulation using Riemannian manifold on pan-cancer data. Unlike most of the existing pathway-based inference tools that calculate the activation of pathways in the Euclidean space, we attempt to introduce a Riemannian manifold-based method to evaluate the pathway activation for each disease sample on the tangent space of the Riemannian manifold. We compare the performance of RiePath and some other feature engineering algorithms, and identify the dysregulated pathways and candidate prognostic pathway biomarkers based on the RiePath scores. The results prove the effectiveness of introducing Riemannian manifold to evaluate the personalized pathway activation for pan-cancer analysis, the effectiveness of generating a relatively low-dimensional and biologically relevant representation, and the robustness of identifying a panel of biologically meaningful pathway signatures as biomarkers. We believe that RiePath has the potential to provide new prospects in understanding the molecular mechanisms of complex diseases and may find broader applications in predicting pathway biomarkers for other intricate diseases.

## Figures and Tables

**Table 1:** Reproducibility comparison measured based on MSE for the five feature engineering algorithms.

Dataset	RiePath	CORGs	GSVA	PLAGE	ssGSEA
BLCA	198.78	0.98	1.04	0.98	196.87
BRCA	352.89	0.97	1.05	0.95	252.27
COAD	286.14	0.93	1.01	0.94	281.74
HNSC	263.76	0.95	1.05	0.97	254.19
KICH	389.52	0.83	1.02	0.77	293.59
KIRC	309.68	0.94	1.00	0.96	297.61
KIRP	387.01	0.91	1.01	0.94	316.69
LGG	688.92	0.94	1.00	0.91	635.00
LIHC	227.58	0.86	1.02	0.89	212.01
LUAD	271.53	0.97	1.03	0.95	242.96
LUSC	308.13	0.97	1.02	0.92	262.08
OV	408.63	0.92	1.02	0.94	358.85
PRAD	771.00	0.87	0.97	0.87	620.76
STAD	235.47	0.97	1.03	0.97	231.12
THCA	683.27	0.91	0.96	0.88	621.84
UCEC	220.73	0.98	1.07	0.97	217.65

**Table 2:** Summary of TCGA datasets for pathway analysis.

Code	Source	Number of Tumor Samples	Number of Normal Samples	Number of Total Samples
BLCA	Bladder carcinoma	400	23	423
BRCA	Breast invasive carcinoma	1057	136	1193
COAD	Colorectal adenocarcinoma	432	54	486
LGG	Low-grade gliomas	509	14	523
HNSC	Head and neck squamous cell carcinoma	496	48	544
PRAD	Prostate adenocarcinoma	428	68	496
THCA	Thyroid carcinoma	504	62	566
KIRC	Kidney renal clear cell carcinoma	523	78	601
KIRP	Kidney renal papillary cell carcinoma	285	32	317
KICH	Kidney chromophobe	64	23	87
LIHC	Liver infiltrate hepatocellular carcinoma	36	5	417
LUAD	Lung adenocarcinoma	499	72	571
LUSC	Lung squamous cell carcinoma	489	52	541
OV	Ovarian carcinoma	358	19	377
STAD	Stomach adenocarcinoma	348	32	380
UCEC	Endometrioid carcinoma	534	32	566

## Data Availability

Data are contained within the article and Supplementary Materials.

## Supplementary Materials

The supporting information can be downloaded at: https://www.mdpi.com/article/10.3390/ijms1010000/s1.
