# Supplementary material for: Pathway Activation Analysis for Pan-Cancer Personalized Characterization Based on Riemannian Manifold
Source: Int J Mol Sci. 2024 Apr 17;25(8):4411. doi: 10.3390/ijms25084411 (PMC11050713; doi:10.3390/ijms25084411)
Supplement: Supplementary file 1 [file ijms-25-04411-s001.zip › Supplementary Figures S1-S15.pdf]

Pathway Activation Analysis for Pan-cancer Personalized Characterization Based on Riemannian Manifold

A

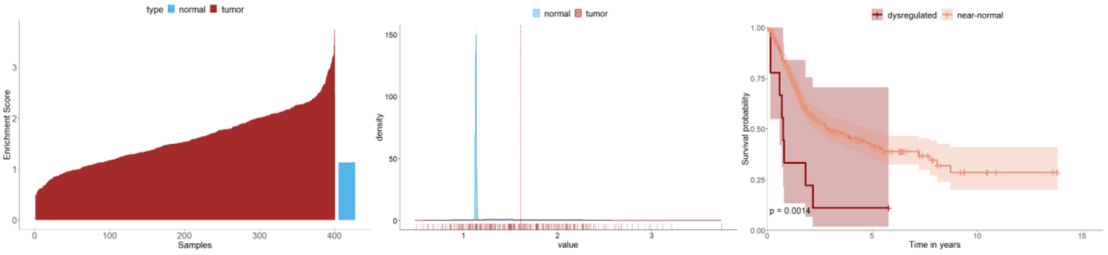

B

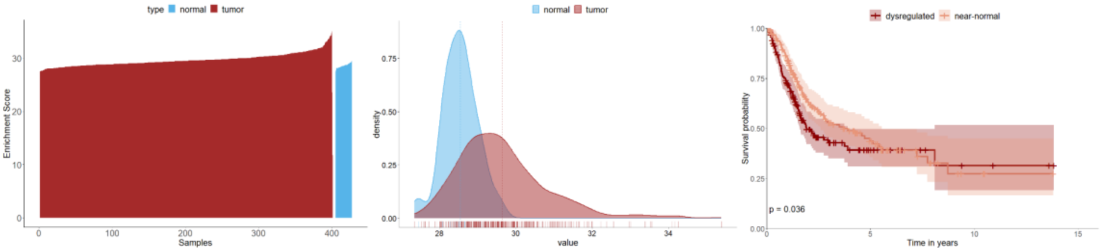

C

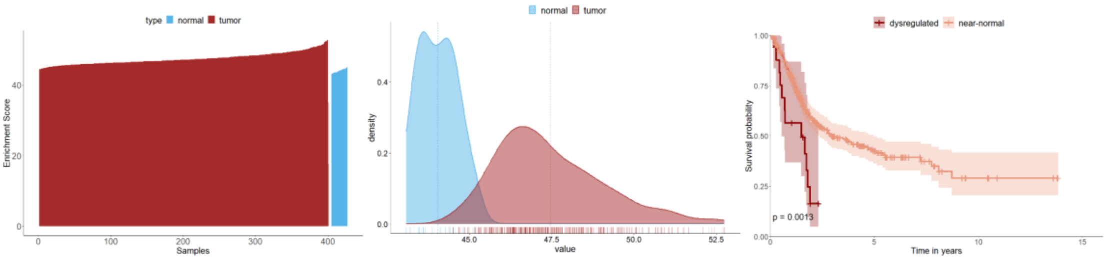

Figure S1. Signaling pathways identified by RiePath scores in BLCA. (A) IL-17 signaling pathway. (B) B cell receptor signaling pathway. (C) HALLMARK_TNFA_SIGNALING_VIA_NFKB.

A

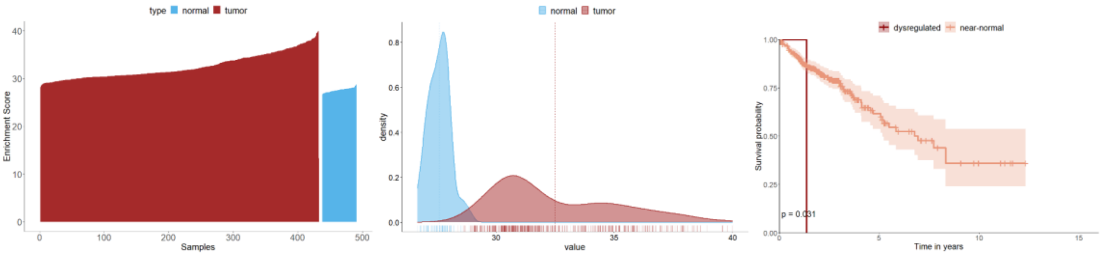

B

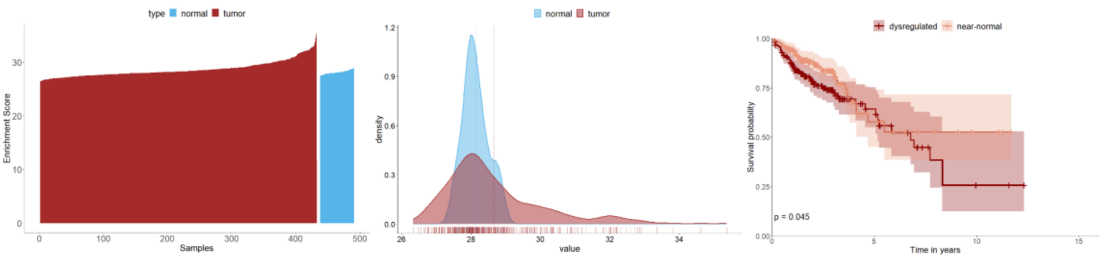

C

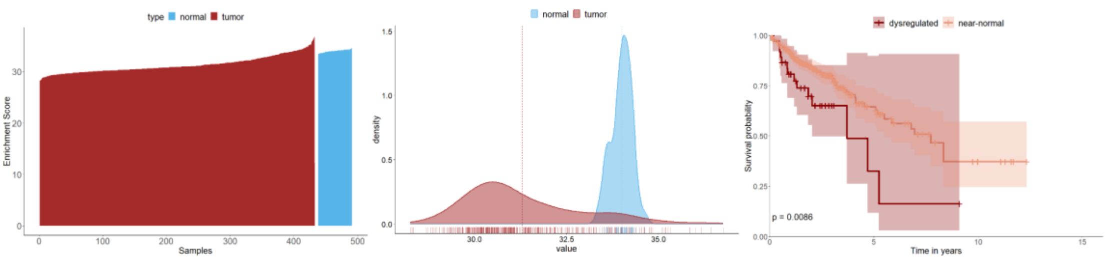

D

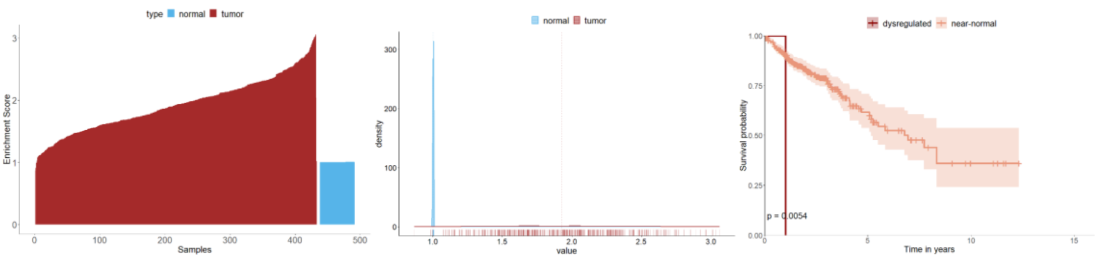

Figure S2. Signaling pathways identified by RiePath scores in COAD. (A) Phospholipase D signaling pathway. (B) C-type lectin receptor signaling pathway. (C) Thyroid hormone signaling pathway. (D) HALLMARK_NOTCH_SIGNALING.

A

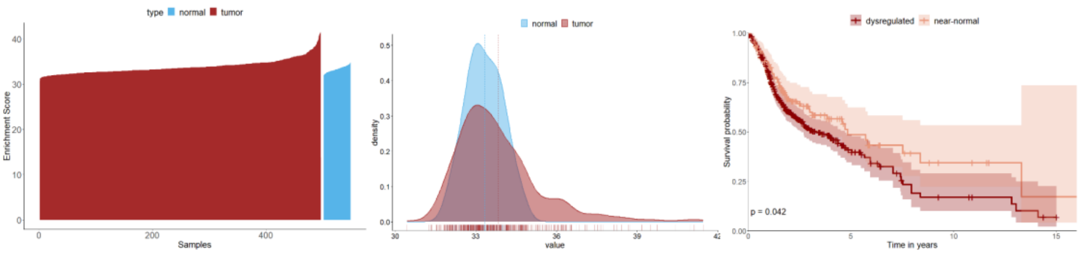

B

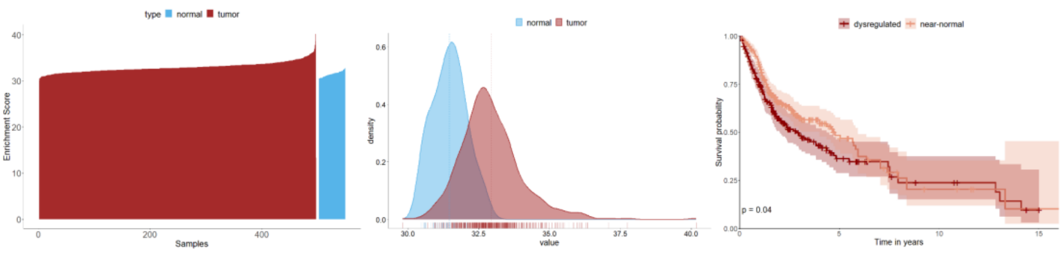

C

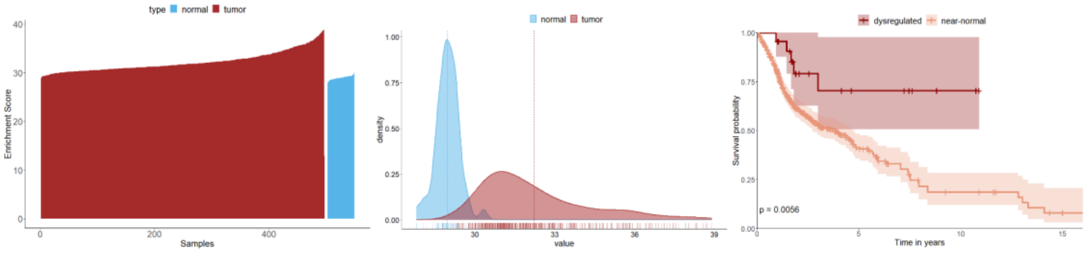

Figure S3. Signaling pathways identified by RiePath scores in HNSC. (A) NOD-like receptor signaling pathway. (B) Oxytocin signaling pathway. (C) HALLMARK_PI3K_AKT_MTOR_SIGNALING.

A

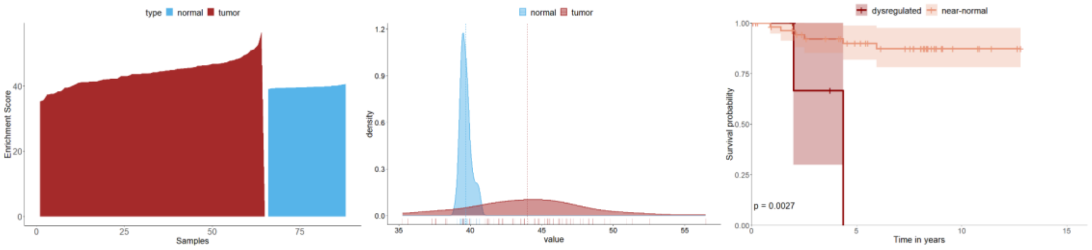

Figure S4. Signaling pathways identified by RiePath scores in KICH. (A) C-type lectin receptor signaling pathway.

A

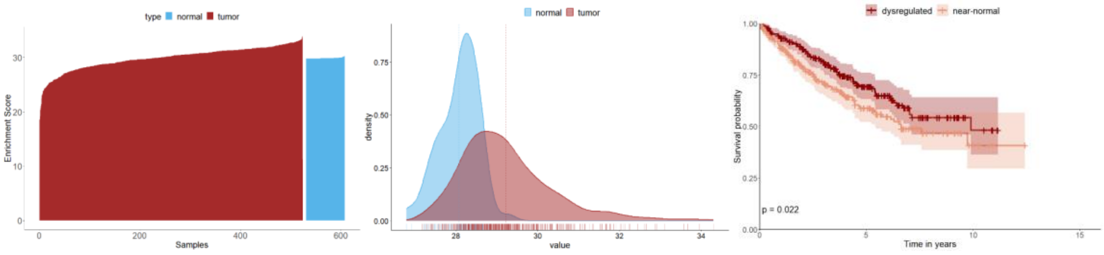

B

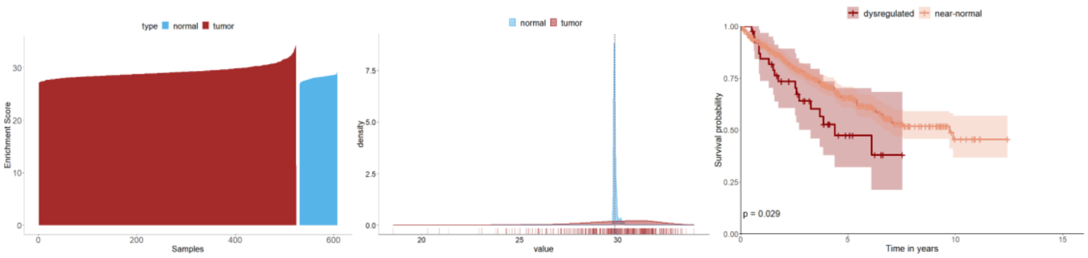

Figure S5. Signaling pathways identified by RiePath scores in KIRC. (A) cGMP-PKG signaling pathway. (B) GnRH signaling pathway.

A

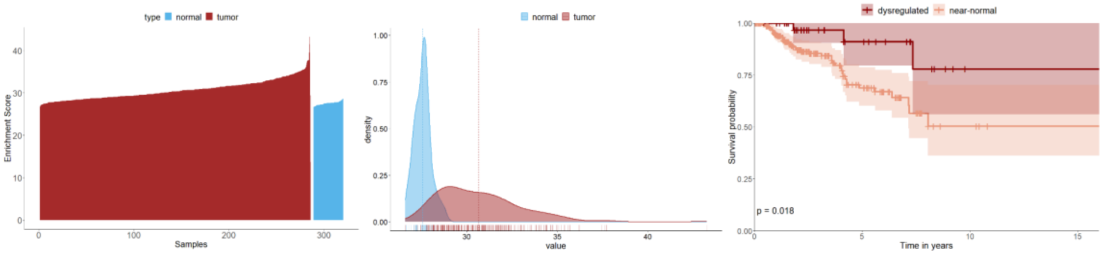

Figure S6. Signaling pathways identified by RiePath scores in KIRP. (A) PPAR signaling pathway.

A

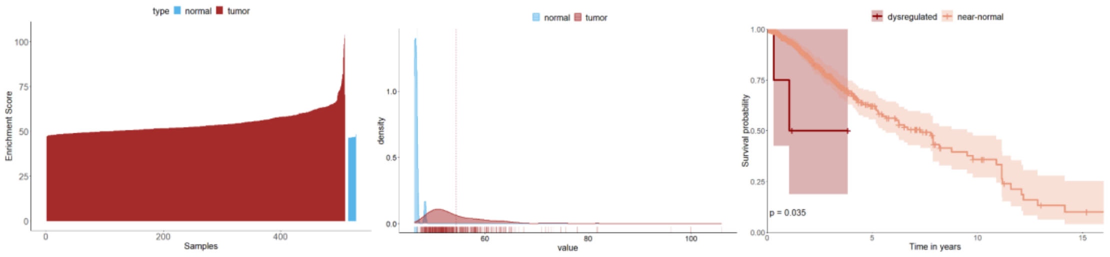

B

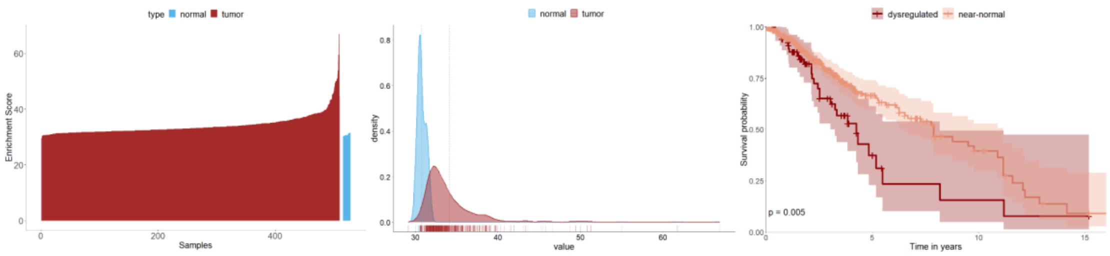

C

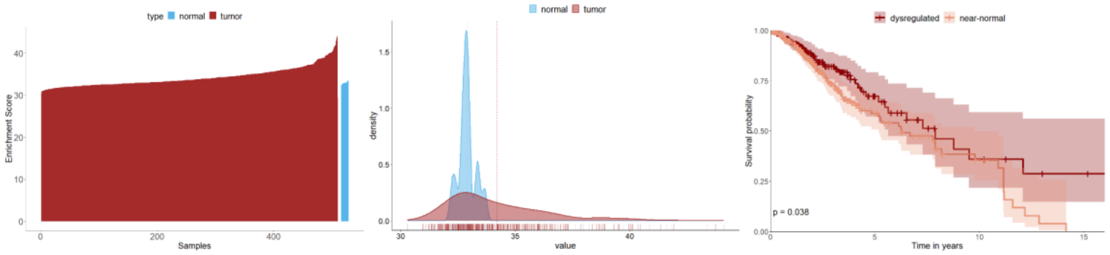

Figure S7. Signaling pathways identified by RiePath scores in LGG. (A) Rap1 signaling pathway. (B) TGF-beta signaling pathway. (C) HALLMARK_IL6_JAK_STAT3_SIGNALING.

A

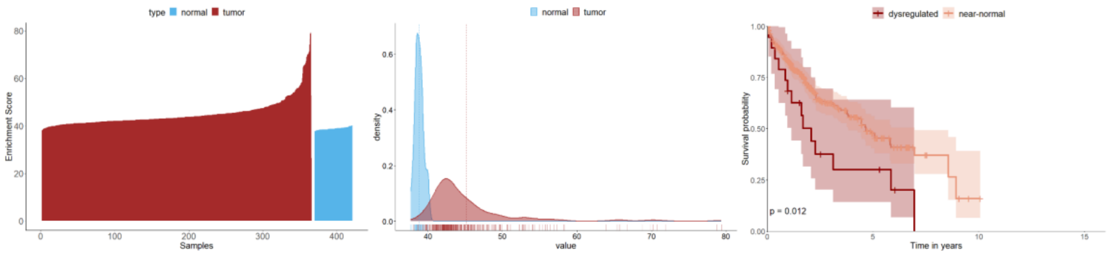

B

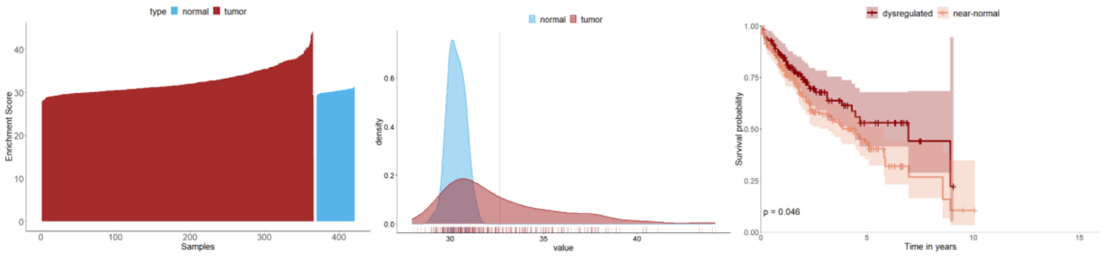

C

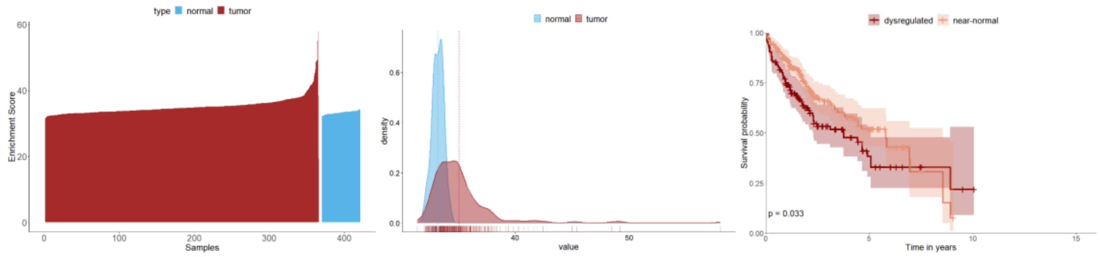

D

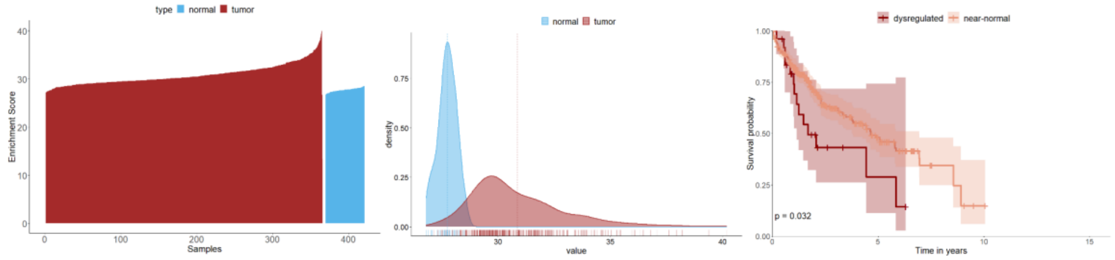

E

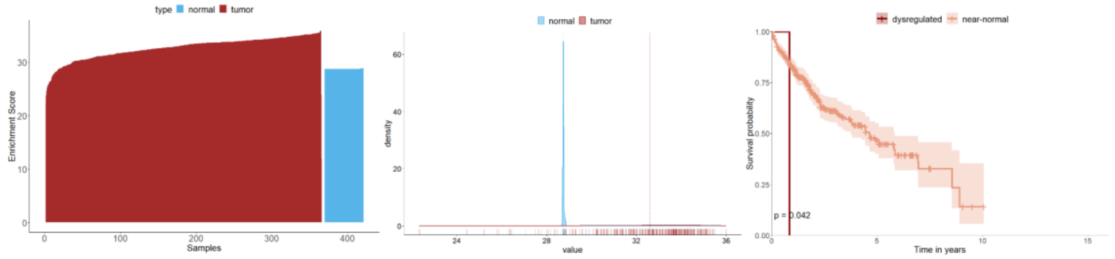

Figure S8. Signaling pathways identified by RiePath scores in LIHC. (A) Ras signaling pathway. (B) Phospholipase D signaling pathway. (C) Hippo signaling pathway. (D) Toll-like receptor signaling pathway. (E) HALLMARK_TGF_BETA_SIGNALING.

A

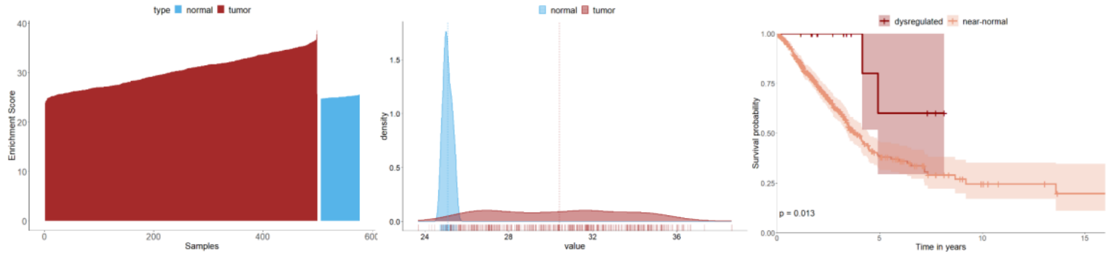

Figure S9. Signaling pathways identified by RiePath scores in LUAD. (A) AMPK signaling pathway.

A

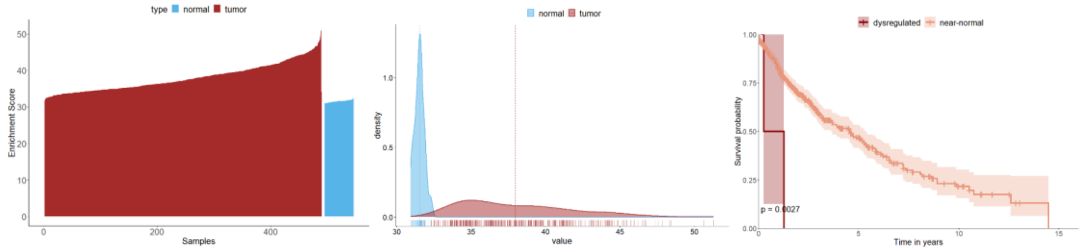

B

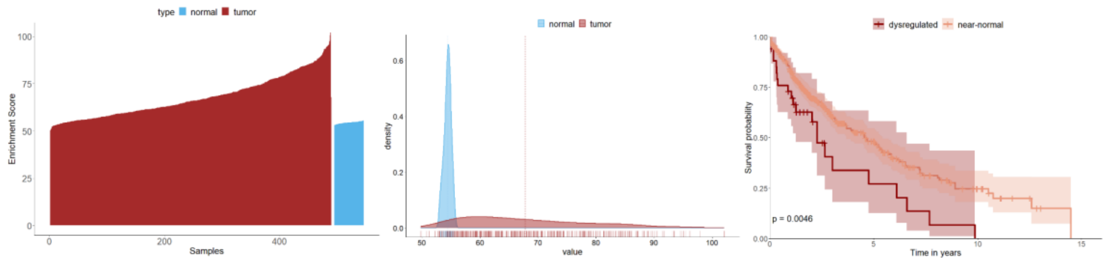

Figure S10. Signaling pathways identified by RiePath scores in LUSC. (A) Apelin signaling pathway. (B) HALLMARK_MTORC1_SIGNALING.

A

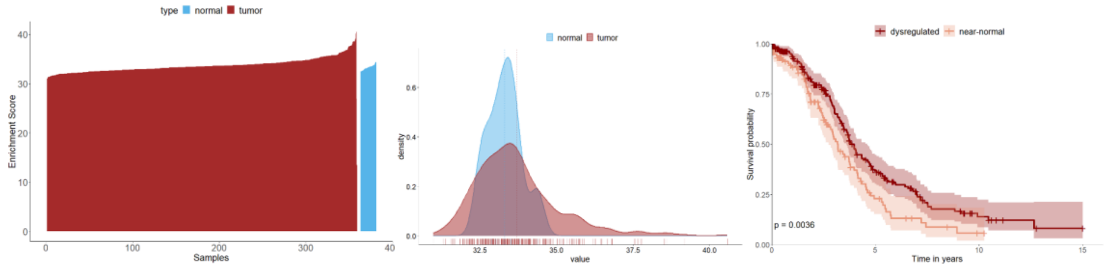

B

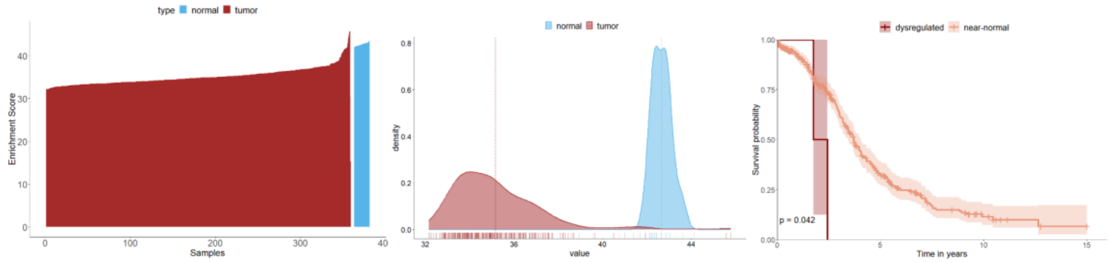

Figure S11. Signaling pathways identified by RiePath scores in OV. (A) HIF-1 signaling pathway. (B) Sphingolipid signaling pathway.

A

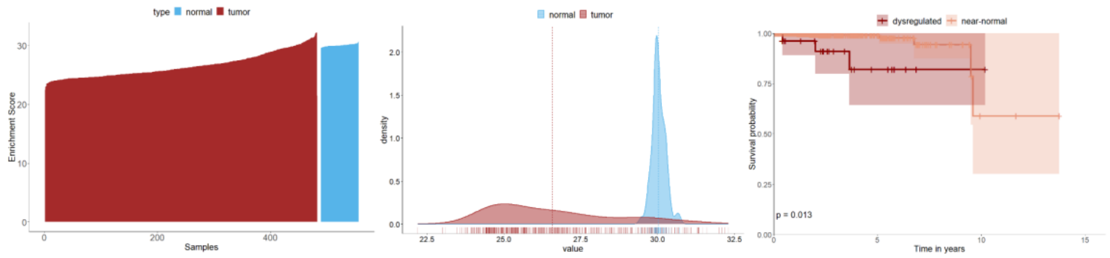

Figure S12. Signaling pathways identified by RiePath scores in PRAD. (A) Retrograde endocannabinoid signaling.

A

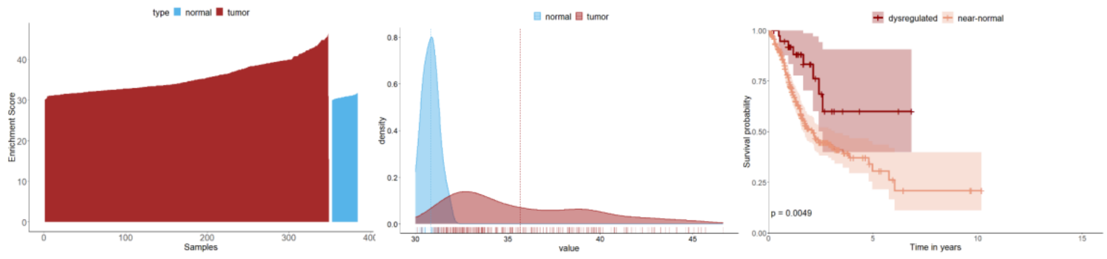

B

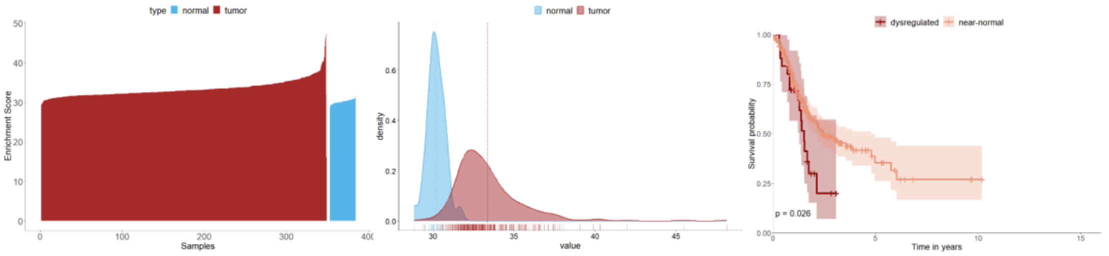

C

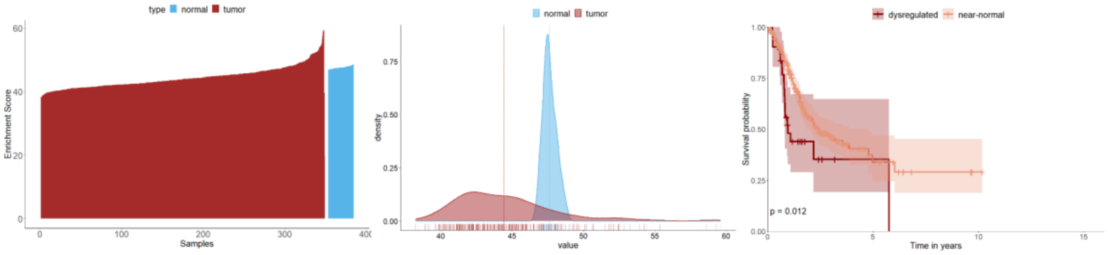

D

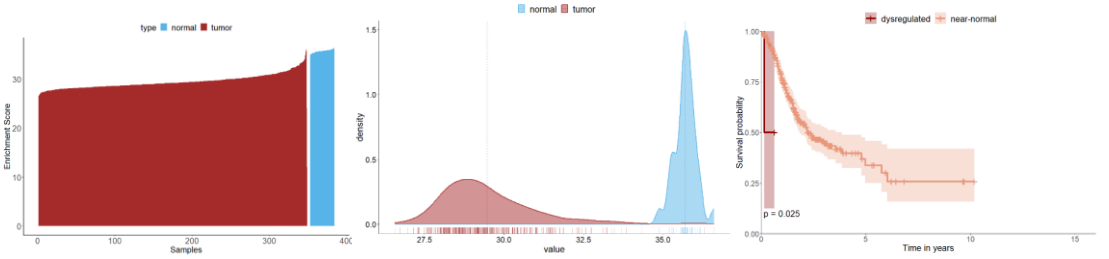

E

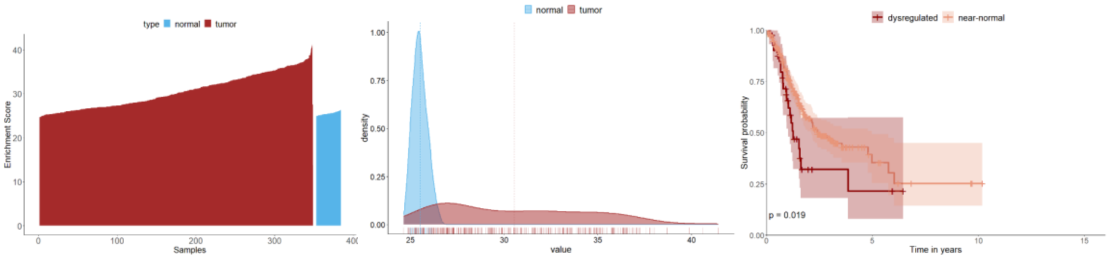

F

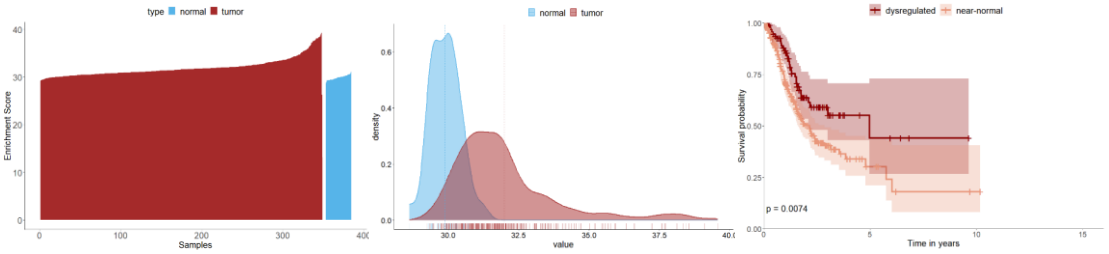

Figure S13. Signaling pathways identified by RiePath scores in STAD. (A) NF-kappa B signaling pathway. (B) HIF-1 signaling pathway. (C) Hippo signaling pathway. (D) TNF signaling pathway. (E) HALLMARK_WNT_BETA_CATENIN_SIGNALING. (F) HALLMARK_IL6_JAK_STAT3_SIGNALING.

A

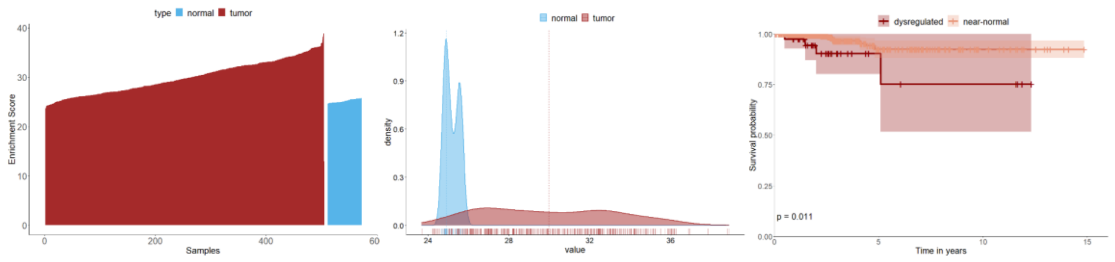

B

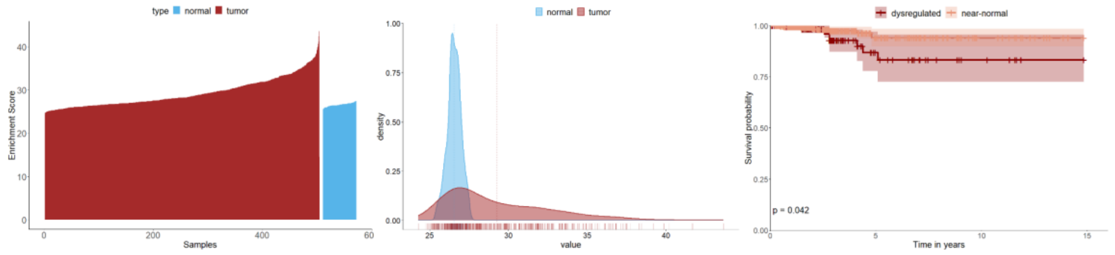

C

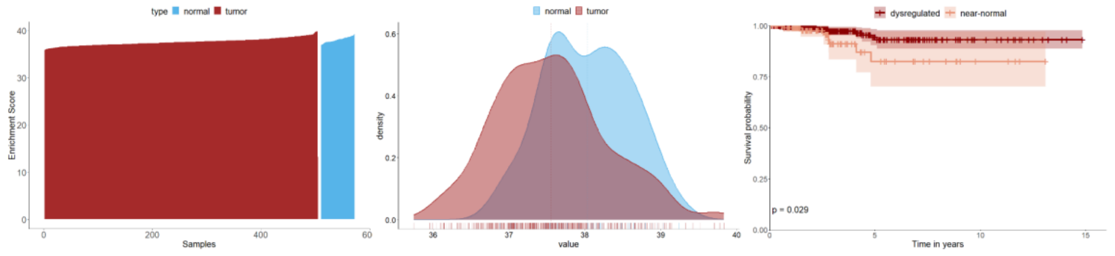

D

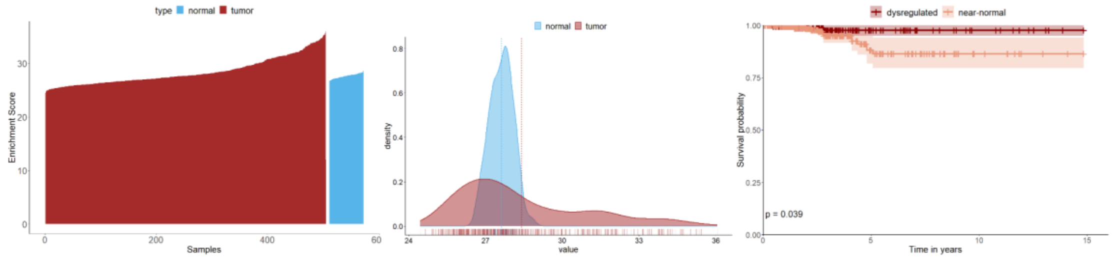

Figure S14. Signaling pathways identified by RiePath scores in THCA. (A) p53 signaling pathway. (B) AMPK signaling pathway. (C) HALLMARK_TNFA_SIGNALING_VIA_NFKB. (D) HALLMARK_IL6_JAK_STAT3_SIGNALING.

A

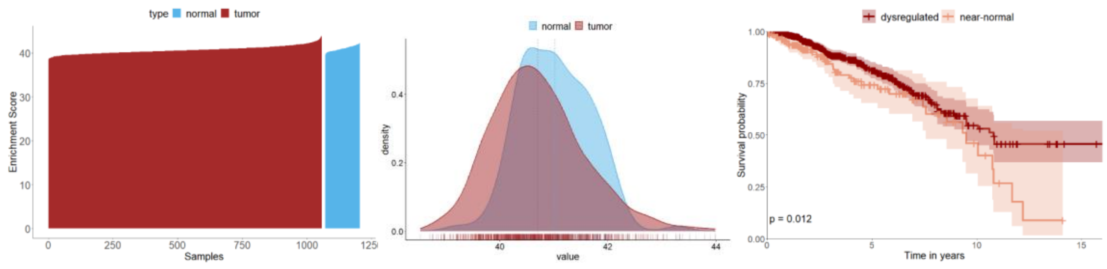

B

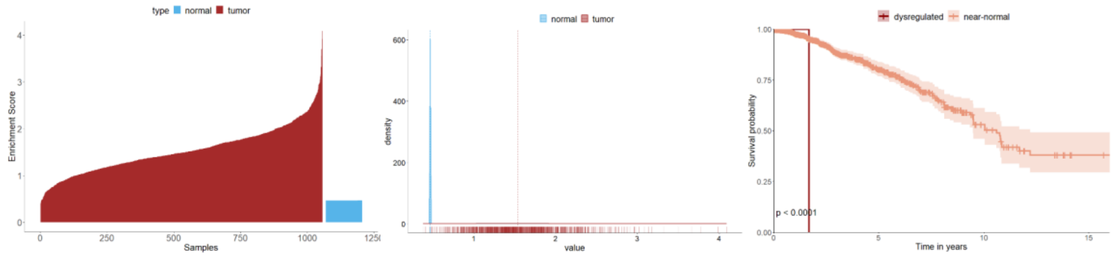

Figure S15. Other two signaling pathways identified by RiePath scores in BRCA. (A) PI3K-Akt signaling pathway. (B) VEGF signaling pathway.
